# COVID-19 vaccination in patients with cancer, a rapid review

**DOI:** 10.3332/ecancer.2022.1355

**Published:** 2022-02-16

**Authors:** Laudy Chehade, Jad Zeitoun, Dorothy Lombe, Sheeba Irshad, Mieke Van Hemelrijck, Karen Canfell, Richard Sullivan, Deborah Mukherji

**Affiliations:** 1 Naef K Basile Cancer Institute, American University of Beirut Medical Center, PO Box 11-0236, Riad El Solh, Beirut 1107 2020, Lebanon; 2 Department of Oncology, Cancer Diseases Hospital, Lusaka 10101, Zambia; 3 Comprehensive Cancer Centre, School of Cancer & Pharmaceutical Sciences, King’s College London, London SE1 9RT, UK; 4 Translational Oncology and Urology Research, King’s College London, London SE1 9RT, UK; 5 Cancer Research Division, Cancer Council NSW, Sydney, NSW 2011, Australia; 6 School of Public Health, University of Sydney, Sydney, NSW 2006, Australia; 7 Prince of Wales Clinical School, University of New South Wales, Kensington 2052, Australia; 8 Institute of Cancer Policy, King’s College London, London, WC2R 2LS, UK

**Keywords:** COVID-19 vaccine, haematologic malignancies, solid organ malignancies

## Abstract

Coronavirus disease 2019 (COVID-19) vaccine development and administration have become global priorities since the beginning of the pandemic, particularly for special populations at higher risk of complications and mortality, such as patients with haematologic and solid organ malignancies. This review aims to summarise the current data for COVID-19 vaccine efficacy in patients with cancer, suggest priority areas for future research and look at potential disparities at a global level. Although patients diagnosed with or receiving therapy for cancer were excluded from the initial vaccine trials, emerging evidence now supports vaccine safety with potentially diminished immune response in this group. Several studies that evaluated antibody response to COVID-19 vaccination found that patients with solid malignancies had lower serologic response rates compared to healthy controls, but better than patients with haematologic malignancies, who had the lowest seroconversion rates and antibody titres. As anticipated, poor serologic responses have been particularly observed among patients receiving B-cell depleting therapies. The data on cellular response are scarce and conflicting since not all studies have showed a difference between patients with malignancies and healthy subjects. Several questions concerning vaccination remain unanswered and require further exploration, such as response duration, need for response monitoring and rates of breakthrough infections.

## Introduction

Since the coronavirus disease caused by the novel severe acute respiratory syndrome coronavirus 2 (SARS-CoV-2) was declared a pandemic by the World Health Organization on 11 March 2020, vaccine development and administration became a global priority. Certain patient populations are more susceptible to developing severe symptoms with a worse prognosis following infection, such as those with an advanced age, cardiovascular disease, diabetes, kidney disease, chronic respiratory disease and others [1–6]. Patients diagnosed with a haematologic or solid organ malignancy are also among the populations who are at greatest risk of death or severe complications of coronavirus disease 2019 (COVID-19) [7–10]. In one study, the case fatality rate was 37% among patients with haematological malignancies and 25% among patients with solid tumours [11]. In a systematic review and pooled meta-analysis of 52 studies, mortality rate from COVID-19 among patients with cancer was found to be 25.6% [12]. The increased COVID-19 complications encountered by patients with cancer may be due to the immunocompromised state resulting from their disease or anti-neoplastic therapy. Vaccination programs initially prioritised patients with advanced age or medical co-morbidities associated with severe infection. However, since patients with cancer were excluded from initial vaccine clinical trials, clear guidance on vaccination in patients with malignancy particularly those on active treatment was relatively slow to be produced due to lack of data. Furthermore, in many countries, especially in low-middle income groups, COVID-19 vaccines were simply not available. The aim of this review is to provide an overview of the current data for COVID19-vaccine efficacy and safety in patients with cancer, suggest priority areas for future research and look at potential disparities at a global level.

### Vaccine development

The Spike S Protein is one of the four structural proteins of the virus, and its major role in viral infectivity makes it a target to most vaccines. It is composed of two subunits; the S1 subunit has the receptor-binding domain and mediates the attachment to the ACE2 receptor on the host cells, and the S2 subunit is responsible for viral fusion with cells [13, 14].

The rapid genomic sequencing of the virus, which became available on 11 January 2020, set the path for fast vaccine development [15].

To the date of this review (27 August 2021), ongoing clinical trials are testing 99 vaccines in humans, with more than 75 preclinical trials experimenting on animals [16]. Some of the vaccines were developed using classical technics and others using more novel technology. Unlike conventional vaccines that are based on actual viral particles, new-generation vaccines contain the protein coding sequence responsible for the synthesis of viral antigenic proteins, which stimulate an immune response [13, 14].

Among classical vaccine platforms, inactivated coronavirus vaccines that have reached phase 3 trials include the BBIBP-CorV, CoronaVac and Covaxin [16]. Several new-generation vaccines that have reached phase 3 trials are being administered world-wide, including messenger RNA vaccines and viral vector-based vaccines. The two leading mRNA vaccines are the mRNA-1273, which is authorised for emergency use in multiple countries, and the BTN162b2 vaccine which was granted full Food and Drug Administration (FDA) approval on 23 August 2021. The mRNA vaccines deliver the mRNA sequence encoding viral spike protein, surrounded by lipid nanoparticles to prevent mRNA degradation. The host cells transcribe the mRNA sequence into the viral protein which is expressed by these cells and presented to the immune cells to produce an immune response [17]. As for viral vector-based vaccines, which deliver the vaccine antigens or genes to the receiver’s cells, ChAdOx1 nCov-19/AZD1222, Gam-COVID-Vac and Ad26.COV2.S have been developed, all of which have received emergency use authorisation in multiple countries [14, 16].

### Vaccination in patients with cancer

Even though COVID-19 vaccines were shown to be effective in preventing hospitalisation and death in registration trials, these trials excluded patients with malignancies who are receiving systemic cytotoxic treatments [18]. Nevertheless, the United States Centers for Disease Control and Prevention (US CDC), National Comprehensive Cancer Network (NCCN), and other professional societies have strongly recommended priority vaccination for immunocompromised individuals including those on active chemotherapy, as these vaccines are not expected to cause major adverse effects and are likely to have a protective effect [19]. Emerging studies have demonstrated good tolerability of the vaccine among patients with cancer, with injection site pain being the most commonly reported side effect [20, 21].

## Literature search

We searched PubMed and MEDLINE on 27 August 2021 to identify clinical trials and observational studies to include in this rapid review (see Appendix A). We used the following keywords: ‘Antibodies, Viral’, ‘Antibodies, Neutralizing’, ‘COVID-19’, ‘SARS Virus’, ‘Immunogenicity, Vaccine’ and ‘Neoplasms’. Additionally, a focused Internet search of the grey literature was done to capture latest updates on guidelines and pre-print articles on vaccine response in patients with cancer. No language restriction was applied to the search. Two authors independently reviewed the list of articles generated by the search and manually selected those that are relevant to the topic and extracted the data. We included studies describing antibody or T-cell response to COVID-19 vaccination in patients with any type of malignancy.

## Findings

### Post-vaccination seroconversion rates of patients with solid organ and haematologic malignancies

The extent to which COVID-19 vaccines are effective in the cancer population is currently being studied. Interim results from the Sars-CoV-2 for Cancer Patients (SOAP-02) study which compared the efficacy of the COVID-19 vaccine BNT162b2 among solid cancer patients, haematological cancer patients, and healthy controls showed that only 38% of patients with solid tumours and <20% of patients with haematological malignancies developed positive anti-S IgG titres 21 days after the first dose, whereas 94% of healthy controls had a positive IgG test [20]. Monin *et al* [20] also studied the effect of administering a second dose and found that in solid cancer patients, 95% of those who received a boost at 3 weeks following the initial vaccination dose developed positive anti-S IgG antibodies 2 weeks later, whereas only 30% of those who did not receive a boost were seropositive. Similar results were found in another study in which only 29% of solid cancer patients who were receiving systemic chemotherapy, immunotherapy, biological agents or a combination of those, were seropositive after the first dose compared to 84% of healthy controls; however, after receiving a second dose, 86% of the patients were seropositive around 2 weeks later [22].

Several studies have looked at the COVID-19 vaccine immune response separately in patient populations with a solid or a haematologic cancer. Patients with a solid cancer had a 94% seroconversion rate (compared to 100% in healthy controls) after two doses of COVID-19 vaccine, and anti-spike antibody titres were significantly low among those on chemotherapy [23]. Similar results were found in two other studies where 90% [24] and 95% [25] of the solid cancer patients developed positive antibody response post second vaccine dose compared to 100% of the heathy controls.

With respect to patients diagnosed with a haematologic malignancy, their seroconversion rates are lower than those of healthy controls. One study demonstrated that while seroconversion rate was 100% in healthy controls 2 weeks post second vaccine dose, it was only 78.6% patients with multiple myeloma and 88% in patients with chronic myeloid leukaemia (CML) and myeloproliferative neoplasms (MPNs) [21]. Other studies found that after two doses of vaccine, 39.5% of patients with chronic lymphocytic leukaemia (CLL) seroconverted versus 100% of healthy controls [26], and 84.2% of patients with multiple myeloma had a positive antibody response versus 100% of the controls [27]. Haematologic malignancy patients were also found to have lower antibody concentrations than healthy controls after two doses of vaccine [28, 29].

Some studies have compared vaccine immune response between patients with a haematologic malignancy and those with a solid organ cancer. Their results have shown that patients with a haematologic cancer have lower rates of seroconversion [20, 30] and lower median antibody titres compared to those with a solid organ cancer [31, 32]. In one study, compared to patients with a solid tumour, patients with a haematological malignancy had lower rates of seroconversion (77% versus 98%) and lower antibody titres (median, interquartile range (IQR): 832 (24–2,500) versus >2,500 (514–2,500)) after two vaccine doses; both these findings were statistically significant [31]. A study by Thakkar *et al* [32] also demonstrated that seroconversion rates were significantly lower in patients with a haematologic malignancy compared to a solid tumour (85% versus 98%), especially in patients on anti-CD20 treatment and stem cell transplantation.

It is worth noting that most of the studies relied on anti-spike antibody measurements as a surrogate marker for immunity against COVID-19, even though the anti-S IgG level that correlates with a clinically relevant viral inhibition is not yet established. The study by Malard *et al* [33], found that an anti-S IgG level above 3,100 UA/mL correlated with >30% neutralising antibodies (NAbs), which is the positive cutoff for NAbs. Moreover, only 46.7% of their cohort tested above this level after two vaccine doses (compared to 87% of the controls). Terpos *et al* [29] considered the neutralising antibody titre cutoff for a positive test to be more than 30%, but a value above 50% to correspond to a clinically relevant viral inhibition. More studies are needed to define clinically significant neutralising antibody and anti-S IgG titre thresholds.

Data on seroconversion rates and antibody titres across different studies are summarised in Tables 1 and 2. It is important to note that the studies followed the standard vaccine schedule that is recommended by the manufacturer, and additional data is needed on the immune response following delayed scheduling.

### Post-vaccination T-cell response in patients with solid organ and haematologic malignancies

Cellular immunity also plays a role in the immune response against COVID-19 [34], and the literature shows discrepant results among studies. T-cell response was assessed across different studies using enzyme-linked immunospot (ELISpot) assay or a variation of it. In brief, cytokines released by the stimulated peripheral blood mononuclear cells (PBMCs) are captured by antibodies attached to a membrane and then detected by using a detection antibody. These tests were used to detect interferon gamma (IFN-γ) and interleukin 2 (IL-2) secreting reactive T-cells in response to COVID-19 vaccination.

Monin *et al* [20] found that following 1 vaccine dose, 82% of controls, 71% of patients with solid tumours and 50% of patients with a haematologic malignancy showed T-cell response. The T-cell response rate in other studies following two vaccine doses was 46% [30] in patients with a solid cancer and 45% [30] to 53% [33] among patients with a haematologic cancer. These results contrast with the findings of the OCTAVE trial, which showed comparable T-cell response rates between healthy controls, patients with solid and haematologic malignancies and haematopoietic stem cell transplant (HSCT) recipients [35].

A positive T-cell response was observed in a number of serologic non-responder patients across several studies [20, 30, 33]. For example, in one study, 17 out of 36 patients who had a T-cell response post-vaccine were seronegative [33], and in another study 26% of seronegative haematologic patients had a T-cell response [30]. Aleman *et al* [36] compared B and T-cell response in healthy volunteers, seropositive and seronegative patients with multiple myeloma. Seronegative patients had lower detection rates of spike protein reactive B-cells and CD4 positive T-cell response. All of these findings indicate that despite the fact that some seronegative patients may develop a T-cell response, a significant portion of these patients also appear to lack cellular immunity against COVID-19.

Table 3 summarises the rates of T-cell response to COVID-19 vaccination in patients with cancer across different studies.

### Effect of treatment types on vaccine response in patients with cancer

The impact of antineoplastic treatment on the COVID-19 vaccine immune response was explored across multiple studies, and some therapies were associated with diminishing the vaccine effect, while others were found not to interfere with the response.

Cytotoxic chemotherapy is among the treatment modalities associated with low rates of seroconversion [23, 30–32]. Similarly, anti-CD20 therapies were associated with low seroconversion rates in several studies [26, 28, 30–33], owing to their B-cell depleting effect and impairment of T-cell dependent and independent response, which can last for up to 12 months [37]. In one study, none of the 22 patients who have received anti-CD20 therapy within 12 months of vaccination mounted an antibody response, in contrast with patients who have completed this treatment more than 12 months before vaccination, out of whom 45.5% developed antibodies [26]. In fact, several authors have advised revaccination for patients on anti-CD20 treatment 6 months after completing their course, or to postpone vaccination till 6 months after the end of treatment [31, 37, 38].

Anti-CD38 treatment, which is used in multiple myeloma, was found to have a negative effect on the immune response. Pimpinelli *et al* [21] showed that patients on proteasome inhibitors or immunomodulatory drugs (imids)-based treatments without Daratumumab had a 92.9% seroconversion rate 2 weeks after the second vaccine dose, compared to 50% in patients on Daratumumab (*p* = 0.003). Similar results were found by Van Oekelen *et al* [27] where anti-CD38 treatment was significantly associated with the risk of having a negative antibody response to the vaccine (*p* = 0.005, odds ratio = 4.258). However, one study did not find a significant association between anti-CD38 therapy and antibody response [30].

Other therapeutic modalities that were also associated with lower seroconversion rates include HSCT, chimeric antigen receptor (CAR-T) cell treatment, Burton tyrosine kinase inhibitors, Ruxolitinib, Venetoclax, steroids and anti-B-cell maturation antigen bispecific antibody therapy [26–28, 30, 32]. AntiPDL1/PD1, anti-CTLA4, hormonal therapy, anti-Epidermal Growth Factor Receptor 2 and antiangiogenic therapy were not found to affect the rates of seroconversion across studies [23, 28, 30–32].

With regard to T-cell response, steroids and being on current treatment for malignancy were associated with lower T-cell response rates in patients with haematologic malignancies [30, 33].

## Areas requiring further research and exploration

### Duration of vaccine response

The duration of the immune response to the COVID-19 vaccine has yet to be explored, as more time passes since the initial vaccine rollout. Ehmsen et al [30] showed a decline in titres and seropositivity rates from 93% at 36 days post-vaccination to 86% at 3 months post vaccination for patients with a solid cancer, and from 66% to 53% for patients with a haematologic malignancy. Only one patient of the cohort, initially seronegative at 36 days, became positive after 3 months. As different vaccine types exist in the market, some being more effective than others, the question of combining different vaccine types to optimise response remains unanswered.

### Heterologous COVID-19 vaccination

The use of different vaccines types for first and second dose has been investigated in some studies that did not involve patients with cancer. The aim of heterologous vaccination is to avoid shortage of one type of vaccine, which might slow down vaccine rollout, while maintaining efficacy. The Com-COV2 study involved 1,072 participants who received BNT162b2 or ChAdOx1 vaccines for their first dose were randomised to receive a homologous vaccine, mRNA-1273 or NVX-CoV2373 vaccine as their second dose after 8 to 12 weeks. The immunogenicity of mRNA-1273 as second dose following ChAdOx1 or BNT162b2 was not inferior to that of homologous vaccine schedule [39]. In another phase II study, BNT162b2 was administered as second dose to participants primed with ChAdOx1, which was shown to result in a strong humoral and cellular response and mild reactogenicity [40]. Further exploration of this topic is needed, with studies involving patients with cancer.

### Third dose recommendation and need for response monitoring

Although vaccination is necessary for patients with cancer, the optimal timing for vaccine administration during treatment and the need for response monitoring remain to be determined. Some authors have recommended assessing vaccine response and to revaccinate after completion of treatment [33, 38]. As of August 2021, the FDA [41], CDC [42] and NCCN [43] have authorised a third vaccine dose for patients with moderate-to-severe immunodeficiencies, including patients with solid and haematologic malignancies on active treatment, CAR-T cell and HSCT recipients, given that these patients might not be able to mount sufficient immunity following the regular vaccine series. The UK Joint Committee on Vaccination and Immunisation has also advised having a third dose part of the primary vaccination series for patients with severe immunosuppression [44]. Although data proving the efficacy of a third vaccine dose in solid organ transplant recipients [45] is available, such data is limited for patients with cancer and may be the topic of future investigations. In a cohort of 20 patients with cancer, 16 patients had improved neutralising antibody titres following a third BNT162b2 vaccine dose, but there was no improvement in circulating spike-specific T cell frequencies [46]. In a recent study, 88 patients with cancer received a COVID-19 booster vaccine 28 days following the standard series. 56% of the patients who were initially seronegative seroconverted after the booster shot (*p* = 0.000062) and those with a haematological malignancy had a smaller change in anti-S IgG titres post booster compared to patients with solid organ tumours (10,034 versus 22,686 AU/mL, *p* = 0.00263) [47].

### Breakthrough infections in patients with cancer and vaccine efficacy against emerging variants

Data are emerging on the rate of breakthrough infections among vaccinated patients with cancer. A study by Heudel *et al* [48] found the rate of infection to be 0.4% in patients who received two doses and 5% in those who took one dose (*p* < 0.0001). 12.5% of the patients who got COVID-19 infection died, making the overall mortality rate 0.7% in patients with a haematologic malignancy and 0.08% in patients with solid tumours. In another prospective cohort study, 9 out of 885 fully vaccinated haematologic malignancy patients had COVID-19 infection; 6 required oxygen supplementation and 3 died [28]. In the study by Van Oekelen *et al* [27] 10 out of 260 patients with multiple myeloma developed COVID-19 infection after receiving at least 1 dose of mRNA vaccine, 3 of which have developed the infection after getting 2 doses. Three of the ten patients required hospitalisation and one patient required intensive care and passed away [27]. It is important to monitor the rate of breakthrough infections, particularly that new variants of COVID-19 are emerging, and the effect of the available vaccines against them is still uncertain.

### Viral shedding in vaccinated patients post infection

Several studies about the duration of viral shedding in patients infected with COVID-19 have been conducted. The main goal of these studies was to determine the approximate number of days during which infected patients must isolate themselves after being diagnosed, in an attempt to decrease the transmission rates. The duration of viral shedding differed based on the severity of the infection. While most infected patients shed infectious particles for a maximum of 8 days [49, 50], some hospitalised patients with high viral loads continue to shed viable viral particles for more than 2 weeks [51, 52]. Patients diagnosed with solid or haematological malignancies are usually on immunosuppressive therapies and therefore tend to shed viral particles for longer periods. Aydillo *et al* [53] showed that cancer patients receiving treatments like HSCT and CAR-T T-cell therapy may shed viable infectious particles for 2 or more months after their initial diagnosis with COVID-19. One immunocompromised patient with CLL continued shedding particles even 70 days post diagnosis [54]. These findings support the CDC guidelines for cancer patients, which state that cancer patients receiving chemotherapy should isolate for a minimum of 20 days after their first positive PCR test [55]. Vaccination against COVID-19 was shown to significantly reduce viral load and shedding in patients with breakthrough infections [56, 57]. However, the literature still lacks data about the effect of vaccines on the viral shedding in immunocompromised patients with solid or haematological malignancies specifically.

## COVID-19 prevention priorities for low-income countries

Currently, vaccination rates are much higher in high-income countries compared to low- and middle-income countries (LMICs) [58]. The continuous widening of the gap in vaccination rates between high-income countries and other groups may be explained by several factors. A recent regression analysis showed that LMICs had significantly lower vaccination coverage and vaccination policy strength relative to high-income countries 6 months after the start of vaccine distribution [59]. In addition, other factors explaining the lower vaccination rates in LMICs may include vaccine hesitancy among the population, lack of awareness, spread of unreliable information about vaccine safety, and low socioeconomic status [60, 61]. Similar factors also seem to play a role in lowering vaccination rates in cancer patients in these countries [62]. Taking all this and the increased susceptibility of cancer patients to higher COVID-19 morbidity and mortality into account, the maintenance of strict precautions in this population of patients in LMICs is extremely important.

## Conclusion

COVID-19 mitigation through vaccination is crucial in patients with solid organ and haematologic malignancies, as they are at high risk of morbidity and mortality from this infection. Despite the fact that patients with cancer were excluded from original vaccine trials, recent studies have showed that these vaccines are safe in this broad population. However, seroconversion is variable and depends on the type of malignancy and treatment, as patients with solid malignancies have lower seroconversion rates than healthy controls, but better than patient with haematologic malignancies. Cellular immunity was also observed to follow a similar trend in some studies, and found to be comparable among these three cohorts in one report. Therefore, a third dose (booster) of the vaccine is likely beneficial for cancer patients, although efficacy data in this population is limited. Response monitoring may also be needed, particularly in patients who are severely immunocompromised. In low and many middle-income countries where the rate and coverage of COVID-19 vaccination is slow, cancer patients will continue to be at higher risk of morbidity and mortality.

## Conflicts of interest

The authors do not report any relevant conflicts of interest.

## Funding

The Cancer and COVID-19 Global Taskforce (https://covidcancertaskforce.org) is funded through UK Research and Innovation as part of the Global Challenges Research Fund; Research for Health in Conflict in the Middle East and North Africa (R4HC-MENA) project, grant number ES/P010962/1.

## Figures and Tables

**Table 1.** Seroconversion rates in patients with cancer following two doses of COVID-19 vaccine.

Study	Location	Vaccine type	Test used	Malignancy type	Seroconversion rate after two vaccine doses			
					Solid cancer % (*n/N*)	Haematologic Malignancy % (*n/N*)	Healthy control % (*n/N*)	*p* value
Massarweh *et al* [24]	Israel	BNT162b2	Anti-S IgG	Solid cancer^a^	90% (92/102)		100% (78/78)	-
Addeo *et al* [31]	USA and Europe	mRNA-1273 or BNT162b2	Anti-S IgG	Solid and haematologic malignancies^b^	98% (99/101)	77% (17/22)	-	0.002
Monin *et al* [20]	UK	BNT162b2	Anti-S IgG	Solid and haematologic malignancies^c^	95% (18/19)	60% (3/5)	100% (12/12)	-
Goshen-Lago *et al* [22]	Israel	BNT162b2	Anti-S IgG	Solid cancer^d^	86% (86/100)	-	-	-
Palich *et al* [23]	France	BNT162b2	Anti-S IgG	Solid cancer^e^	94% (210/223)	-	100% (49/49)	-
Thakkar *et al* [32]	USA	mRNA-1273, BNT162b2 or AD26.COV2.S	Anti-S IgG	Solid and haematologic malignancies^f^	98% (131/134)	85% (56/66)	-	0.001
Ehmsen *et al* [30]	Denmark	mRNA-1273 or BNT162b2	Anti-S IgG	Solid and haematologic malignancies^g^	93% (187/201)	66% (215/323)	-	0.004
Malard *et al* [33]	France	BNT162b2	Anti-S IgG	Haematologic malignancies^h^	-	46.7% (195)	87% (30)	0.0002
Van Oekelen *et al* [27]	USA	mRNA-1273 or BNT162b2	Anti-S IgG	Multiple myeloma	-	84.2% (219/260)	100% (67/67)	-
Lim *et al* [38]	UK	ChAdOx1 or BNT162b2	Anti-S IgG	Lymphoma^i^	-	-	-	-
Pimpinelli *et al* [21]	Italy	BNT162b2	Anti-S IgG	Multiple myeloma and myeloproliferative malignancies	-	MM: 78.6% (33/42) MPM: 88% (44/50)	100% (36/36)	MM: 0.003 MPM: 0.038
Herishanu *et al* [26]	Israel	BNT162b2	Anti-S IgG	CLL	-	52% (27/52)	100% (52/52)	<0.001
Barrière *et al* [25]	France	BNT162b2	Anti-S IgG	Solid tumours	95.2% (40/42)	-	100% (24/24)	-
Kearns *et al* [35]	UK	BNT162b2 or ChAdOx1	Anti-S IgG	Immunocompromised patients^j^	100% (47/47)	88.9% (16/18) in patients with haematologic malignancies and 88.1% (37/42) in HSCT patients^k^	100% (93/93)	-
Roeker *et al* [63]	USA	BNT162b2 or mRNA-1273	Anti-S IgG	CLL	-	52% (23/44)	-	-
Terpos *et al* [29]	Greece	BNT162b2 or ChAdOx1	NAbs against SARS-CoV-2	Multiple myeloma, smoldering myeloma, and monoclonal gammopathy of undetermined significance (MGUS)	-	Neutralising effect ≥ 30%: 71% (196/276) Neutralising effect ≥ 50%: 57.3% (158/276)	Neutralising effect ≥ 30%: 90.3% (204/226) Neutralising effect ≥50%: 81% (183/226)	<0.001
Parry *et al* [64]	UK	BNT162b2 or ChAdOx1	Anti-S IgG	CLL	-	Serum samples: 75% (9/12) Dried blood spot samples: 71% (39/55)	Serum samples: 100% (59/59) Dried blood spot samples: 97% (36/37)	-
Benjamini *et al* [65]	Israel	BNT162b2	Anti-S IgG	CLL	-	43% (160/373)	-	-

a Gastrointestinal, lung, breast, brain, genitourinary cancers and others

b Breast, urologic, gynaecologic, skin, thoracic, gastrointestinal, head and neck, brain and connective tissue cancers. Acute lymphoblastic leukaemia (ALL), CLL, chronic myeloid leukaemia (CML), diffuse large B-cell lymphoma (DLBCL), follicular lymphoma, mucosa-associated lymphoid tissue (MALT) lymphoma, T-cell lymphoma/mycosis fungoides, Hodgkin lymphoma (HL), polycythaemia vera, myeloma

c Gynaecological, breast, renal, prostate, testicular, bladder, skin, thoracic, gastrointestinal and head and neck cancers, glioblastoma, CLL/small lymphocytic lymphoma (SLL), plasma cell myeloma, DLBCL, follicular lymphoma, Burkitt’s lymphoma, lymphoplasmacytic lymphoma, mantle cell lymphoma, MALT lymphoma, nodular sclerosing HL, post-renal transplant lymphoproliferative disorder, anaplastic large cell lymphoma, angioimmunoblastic T-cell lymphoma, acute myeloid leukaemia (AML), myelodysplastic syndrome (MDS) or MPN, CML, T-cell precursor ALL, myelofibrosis

d Gastrointestinal, breast, genitourinary, gynaecologic, head and neck, lung, melanoma, neurologic and sarcoma

e Breast, digestive, lung, gynaecological, prostate, bladder, pancreas, kidney, upper aero-digestive tract cancers

f Breast, gastrointestinal, genitourinary, gynaecologic, thoracic, head and neck, skin/musculoskeletal, carcinoma of unknown primary, lymphoid, myeloid and plasma cell malignancies

g Breast, gastrointestinal, urological, gynaecologic, thoracic, skin cancers, CLL/SLL, multiple myeloma, DLBCL, follicular lymphoma, mantle cell lymphoma, marginal zone lymphoma, others

h ALL, non-HL, HL, CLL, multiple myeloma, MGUS, AML, MDS, MPN, others

i HL, aggressive B-cell non-HL, indolent B-cell non-HL, natural killer T-cell lymphoma

j Tumour necrosis factor alpha, IL-2 and granulocyte macrophage colony-stimulating factor

k Patients who underwent HSCT were analysed separately from patients with haematologic malignancies

**Table 2.** Antibody titers in patients with cancer following two doses of COVID-19 vaccine.

Study	Location	Vaccine type	Test type	Malignancy type	Antibody titers, median (IQR) (range)			
					Solid cancer	Haematologic malignancy	Healthy controls	*p* value
Massarweh *et al* [24]	Israel	BTN162b2	Anti-S IgG	Solid cancer	1,931 (509–4,386) (0.3–53,088)		7,160 (3,129–11,241) (442–27,568)	<0.001
Lim *et al* [38]	UK	ChAdOx1 or BTN162b2	Anti-S IgG	Lymphoma	-	2.5 BAU/mL (95% CI 1.1–5.8) for participants on treatment and 141.8 BAU/mL (75.6–266.0) for participants not on treatment.	2,339 BAU/mL (1,923–2,844) in those who took BNT162b2; 199 BAU/mL (140–282) in those who took ChAdOx1	-
Addeo *et al* [31]	USA and Europe	mRNA-1273 or BNT162b2	Anti-S IgG	Solid and haematologic malignancies^a^	>2,500 (514–2,500)	832 (24–2,500)	-	0.029
Palich *et al* [23]	France	BNT162b2	Anti-S IgG	Solid cancer^b^	252 (Roche Elecsys assay); 4,443 Abbott Alinity Assay	-	2,517 (Roche Elecsys assay); 13,285 Abbott Alinity assay	<0.01 for both assays
Thakkar *et al* [32]	USA	mRNA-1273, or BNT162b2 or AD26.COV2.S	Anti-S IgG	Solid and haematologic malignancies^c^	7,858	2,528	-	0.013
Van Oekelen *et al* [27]	USA	mRNA-1273 or BNT162b2	Anti-S IgG	Multiple myeloma	-	149 (5–7,882 AU/mL)	300 (21–3,335 AU/mL)	<0.0001
Pimpinelli *et al* [21]	Italy	BNT162b2	Anti-S IgG	Multiple myeloma and myeloproliferative malignancies	-	MM: 106.7 AU/mL MPM: 172.9 AU/mL	353.3 AU/mL	MM: 0.003 MPM: 0.049
Herishanu *et al* [26]	Israel	BNT162b2	Anti-S IgG	CLL	-	0.824 U/mL	1,084 U/mL	<0.001
Barrière *et al* [25]	France	BNT162b2	Anti-S IgG	Solid tumours	245.2 UI/mL	-	2,517 UI/mL	<0.001
Maneikis *et al* [28]	Lithuania	BNT162b2	Anti-S IgG	Haematological malignancies	-	6,961 AU/mL	21,395 AU/mL	<0.0001
Kearns *et al* [35]	UK	BNT162b2 or ChAdOx1	Anti-S IgG	Immuno compromised patients^d^	4,101 (655–10,819 U/mL)	1,011.5 (17.3–3,877 U/mL) in patients with haematologic malignancies and 980 (91.9–5,129 U/mL) in HSCT patients^e^	11,514 (3,324–23,302 U/mL)	-
Parry *et al* [64]	UK	BNT162b2 or ChAdOx1	Anti-S IgG	CLL	-	53 U/mL	3,900 U/mL	<0.0001

a Breast, urologic, gynaecologic, skin, thoracic, gastrointestinal, head and neck, brain and connective tissue cancers. ALL, CLL, CML, DLBCL, follicular lymphoma, MALT lymphoma, T-cell lymphoma/mycosis fungoides, HL, polycythaemia vera, myeloma

b Breast, digestive, lung, gynaecological, prostate, bladder, pancreas, kidney, upper aero-digestive tract cancers

c Breast, gastrointestinal, genitourinary, gynaecologic, thoracic, head and neck, skin/musculoskeletal, carcinoma of unknown primary, lymphoid, myeloid and plasma cell malignancies

d Tumour necrosis factor alpha, IL-2 and granulocyte macrophage colony-stimulating factor

e Patients who underwent HSCT were analysed separately from patients with haematologic malignancies

**Table 3.** T-cell response in patients with cancer following 2 doses of COVID-19 vaccine.

Study	Location	Vaccine type	Test type	Malignancy type	T-cell response rate						
					Solid	Haematologic		Healthy control			*p* value
Monin *et al* [20]	UK	BNT162b2	Fluorospot assay for IFNγ-producing and IL-2-producing SARS-CoV-2-reactive T-cells	Solid and haematologic malignancies^a^	88% (14/16)	75% (3/4)		100% (3/3)			-
Ehmsen *et al* [30]	Denmark	mRNA-1273 and BNT162b2	Whole blood IFN-γ release immunoassay	Solid and haematologic malignancies^b^	46% (92/201)	45% (144/323)		-			-
Malard *et al* [33]	France	BNT162b2	IFN-γ production measurement on PBMCs (ELISPOT assay)	Haematologic malignancies^c^	-	53% (36/68)		-			-
Aleman *et al* [36]	USA	mRNA-1273 and BNT162b2	IFN-γ, TNF-α, IL-2 and GM-CSF^d^ measurement within CD4+ and CD8+ T-cells through intracellular cytokine staining	Multiple myeloma	-	CD4+ response in 96% (25/26) of seropositive patients and 35% (6/17) of seronegative patients		100%			<0.001
Study	**Location**	**Vaccine type**	**Test type**	**Malignancy type**	**IFN-** γ** secreting T-cell/10^6^ PBMCs, median (IQR)**						
Kearns *et al* [35]	UK	BNT162b2 and ChAdOx1	Spike specific IFN-γ T cell response (Oxford Immunotec T-SPOT Discovery SARS-CoV-2 assay)	Immunocompromised patients^e^	32% (8–112)		54 (20–164) in patients with haematologic malignancies and 32 (8–108) in HSCT patients		60% (20–136)	-	

a Gynaecological, breast, renal, prostate, testicular, bladder, skin, thoracic, gastrointestinal and head and neck cancers, glioblastoma, CLL/SLL, plasma cell myeloma, DLBCL, follicular lymphoma, Burkitt’s lymphoma, lymphoplasmacytic lymphoma, mantle cell lymphoma, MALT lymphoma, nodular sclerosing HL, post-renal transplant lymphoproliferative disorder, anaplastic large cell lymphoma, angioimmunoblastic T-cell lymphoma, AML, MDS or MPN, CML, T-cell precursor ALL, myelofibrosis

b Breast, gastrointestinal, urological, gynaecologic, thoracic, skin cancers, CLL/SLL, multiple myeloma, DLBCL, follicular lymphoma, mantle cell lymphoma, marginal zone lymphoma, others

c ALL, non-HL, HL, CLL, multiple myeloma, MGUS, AML, MDS, MPN, others

d Tumour necrosis factor alpha, IL-2 and granulocyte macrophage colony-stimulating factor

e Inflammatory arthritis, antineutrophil cytoplasmic antibodies-associated vasculitis, end stage kidney disease patients requiring with or without immunosuppression, hepatic disease, inflammatory bowel disease, solid cancer, haematologic malignancy, HSCT

## Appendix A. Literature search strategy in MEDLINE.

1. exp *Antibodies, Neutralising/bl, im [Blood, Immunohalogy]
2. COVID-19 Vaccines/ae, bl, im, tu, to [Adverse Effects, Blood, Immunology, Therapeutic Use, Toxicity]
3. exp *Antibodies, Viral/bl, im [Blood, Immunology]
4. exp *COVID-19/bl, im, pc [Blood, Immunology, Prevention & Control]
5. exp *SARS Virus/im [Immunology]
6. exp *Neoplasms/
7. 1 or 2 or 3 or 4 or 5
8. 6 and 7
9. exp *Immunogenicity, Vaccine/de, im [Drug Effects, Immunology]
10. 7 or 9
11. 6 and 10
